# Undernutrition and its associated factors among pregnant mothers in Gondar town, Northwest Ethiopia

**DOI:** 10.1371/journal.pone.0215305

**Published:** 2019-04-22

**Authors:** Abel Fekadu Dadi, Hanna Demelash Desyibelew

**Affiliations:** 1 Department of Epidemiology and Biostatistics, Institute of Public Health, College of Medicine and Health Sciences, University of Gondar, Gondar, Ethiopia; 2 Department of Public Health Nutrition, School of Public Health, College of Medicine and Health Sciences, Bahir Dar University, Bahir Dar, Ethiopia; Sefako Makgatho Health Sciences University, SOUTH AFRICA

## Abstract

**Background:**

Regardless of significant gains and signs of progress in the last decades, maternal undernutrition remains a major public health concern in Ethiopia. Supporting the progress of interventions being taken in the country with evidence might be important to keep the sustainability of the government effort. We aimed at determining the extent of undernutrition and its associated factors among pregnant mothers in Gondar town, Northwest Ethiopia.

**Method:**

A community-based cross-sectional study was conducted by including 940 selected pregnant mothers through a cluster sampling. A face-to-face interview was administered to pregnant mothers at a household level. We collected data using an Online Data collection kit (ODK) and the collected data was directly downloaded from the Google Cloud platform and finally imported to Stata 14 for further analysis. A multivariable logistic regression model was fitted to identify factors associated with undernutrition. A crude and adjusted odds ratio with their 95% confidence interval was calculated to declare the association and its significance. Model fitness was assured through the *Hosmer* and *Lemeshow* goodness of fit test and model classification accuracy.

**Result:**

14.4% (95%CI: 12.3–16.7) of pregnant mothers were undernourished. After adjusting for the main covariates; as the age of the pregnant mothers increases the odds of being undernourished decreases by 10% (AOR: 0.90; 95%CI: 0.87–0.95) and having a poor marital condition (AOR: 2.18; 95%CI: 1.03–4.59) increased the odds of undernutrition. The risk of undernutrition was also decreased by 43% among those pregnant mothers who consumed coffee sometimes (AOR: 0.57; 95%CI: 0.36–0.89) as compared to daily consumers.

**Conclusion:**

A significant proportion of pregnant mother were undernourished. Integration of nutritional interventions with maternity health services would be highly important to improve the nutritional status of the mothers. It is also important to counsel pregnant mothers about a consequence of frequent coffee drinking during their pregnancy.

## Background

Globally, malnutrition is an important health concern, predominantly in under-five children and pregnant women. The World Health Organization(WHO) classifies malnutrition as the greatest threat to public health [1] and every country is facing a serious challenges from malnutrition [2, 3]. In spite of extensive global economic growth in recent decades, maternal undernutrition is highly prevalent in most countries in south-central and southeastern Asia and Sub-Saharan Africa [3–6].

Ethiopia is one of the countries with a high burden of maternal and child undernutrition. Though, maternal undernutrition has declined over the past 16 years, from 30% in 2000 to 22% in 2016, Ethiopia is still among countries with a high burden of maternal malnutrition [7]. Specifically, two institution based cross-sectional studies conducted in Amhara region reported a prevalence rate of undernutrition ranging from 16% to 29.8% that also showed a significant geographic variation [3, 8].

Maternal undernutrition in the low and middle-income countries is an underlining cause for a 3.5 million mother’s deaths and disabilities due to physical and mental effects of poor dietary intake in the earliest months of life [7, 9, 10]. Previous studies have established that undernourished pregnant women suffer from a combination of chronic energy deficiency that leads them to have a low birth weight (LBW), preterm and unsuccessful birth outcomes [11–13].

Regardless of a significant gains and signs of progress in the last decade, maternal undernutrition still remains a major public health problem in Ethiopia [3, 14]. The government of Ethiopia has developed a revised national nutrition program in 2016 to address the double burden of malnutrition in pregnant and lactating women [1, 10, 15]. Even though a progress of this program implementation needs to be supported with a continuous evidence through research, a limited institution based studies that lack an important variables crucial for prioritizing, designing and initiating intervention programs have been conducted [8]. The objective of this study was, therefore, to assess the magnitude of undernutrition at the community level by including an important variables among pregnant women living in Gondar town.

## Methods

### Study setting, design, and population

We conducted a cross-sectional study from June 15 to July 30, 2018, on pregnant mothers in their second and third trimester of pregnancy who are living in Gondar Town. Gondar town is located in the Northern part of Amhara regional state at a distance of 747 km away from Addis Ababa and 170 km from Bahir Dar (the regional capital city). Gondar town has a total population of 333,103 and an expected number of pregnant women in the town is estimated to be 11,225 in which at least 8,913 of them are living in urban kebeles (clusters) in 2017/2018. Pregnant women who were living in a randomly selected urban kebeles were considered as the study population.

### Sample size and participant recruitment

A cluster sampling was used to reach the study participants. On the first stage, five urban clusters from 12 urban clusters were selected by lottery method and on the second stage, a house to house census of pregnant mothers found in the six selected clusters was conducted. The required sample size was determined in Epi Info 7 by using a single and double population proportion formula. A parity variable from a study conducted to determine undernutrition in Gondar referral hospital [8] was used to determine a sample size for our study assuming 80% power, 95% confidence level, an odds ratio of 2.25, proportion of undernutrition in those women’s with no previous birth as 11.62%, proportion of undernutrition among pregnant mothers with >4 births as 22.8%, cluster effect of 2, and a 10% non-response rate. Our double population proportion formula yielded the higher sample size of 858. However, because of the nature of the cluster sampling, 940 pregnant mothers were actually found and included in the study.

### Data collection

An interviewer-administered Amharic version of the questionnaire was used to collect the required information from the study participants. The online data collection kit (ODK) application was used to collect and manage data to improve its quality. The prepared questionnaire was designed on the excel spreadsheet, converted to XLSForm online, and checked for its validity using Enketo. The validated form was downloaded and uploaded on a Lenovo tab 7 ODK application. The data storage place for the project was created on the Google cloud platform. The data collectors sent the collected data to the online created data storage system and the principal investigator directly downloaded the data from the system.

Maternal nutrition was assessed using a Mid Upper Arm Circumference (MUAC) and categorized as undernutrition (MUAC < 22) and normal (MUAC > = 22) [8, 16]. Maternal depression and anxiety were measured by using an Edinburgh Postnatal Depression Scale (EPDS) revised for Ethiopian context [17]. A mother was considered as depressed if she had a total measurement scale of > = 12. Anxiety was measured by using the third, fourth, and fifth scale on EPDS and a mother with a total scale of > = 6 was classified as having anxiety symptoms [18]. Social support was assessed by using the Oslo Social Support Scale (OSSS-3) and pregnant mothers who scored nine and above were labeled as having “Good” social support and those scored below nine were labeled as having “Poor” social support [19].

Husband support was assessed by a question “*My husband helps me a lot*” with the options: “*Always (5)”*, *“Most of the time (4)*, *“Some of the time (3)”*, *“Rarely (2)”*, *and “Never (1)*". Coffee consumption was assessed based on the number of days the mother consumed coffee in a week. Those who have been consuming coffee one to three days per week were considered as consuming coffee sometimes, those reported consuming coffee every day as daily drinkers, and those have not been drunk before as non-drinkers. A marital condition was also assessed based on mother's perspective regarding their marital situation in a day to day life and if the marital situation is loving and easy going without conflict and disagreement was considered as "Bad" and if not it was considered as “Good”. Physical activity of the mother was assessed by a question “*Have you practice physical activity such as brisk walking*, *dancing*, *gardening*, *and usual housework for at least three hours/week*” and their answer was documented as “Yes” and “No” [20]. Food access for the last three months was assessed by a single question: “*In the last three months*, *have you ever worried that your household would not have enough food*?” a standard question which has been used for assessing the level of food inaccessibility in a household.

The data collection tool was first prepared in English, translated into Amharic and back-translated to English to check for its consistency before administration. Nine trained BSc. nurses were recruited, trained, and collected the data through a house to house survey. The principal investigator and one additional recruited field supervisor supervised the overall data collection activity.

### Data analysis

A data that was collected online was downloaded from the Google Cloud Platform and imported to the Stata 14 for further analysis. Data were checked and re-checked for completeness before importing and further cleaning was done by running frequencies. Mean, median, proportion/percentage, interquartile range, standard deviations, and exploratory analysis were conducted to understand the nature of the data. Preliminary findings were presented using tables. A bi-variable and multivariable logistic regression model was fitted to identify factors associated with undernutrition. Adjusted odds ratio with its 95% confidence interval was computed to test for statistical significance. Model adequacy was checked using the *Hosmer* and *Lemeshow* goodness of fit test (p-value = 0.59) and a classification accuracy (85.6%).

### Ethical consideration

The University of Gondar Institutional Review Board ethics committee approved this study. A support letter was obtained from the University of Gondar Research and Community Service to the Gondar town health office and respective districts. Participants of the study were informed about the purpose, objectives and their right to participate or not participate in the study. Privacy and confidentiality of the study participant were ensured by not using a personal identifier. Written informed consent was obtained from the study participants in order to be part of the study. Pregnant mothers who were seriously ill during a house to house data collection time were referred to Gondar University Specialized Hospital and those found severely malnourished were also counseled about proper nutrition.

## Result

### Socio-demographic and maternal characteristics of the participants

A total of 940 pregnant mothers participated in the study. Most of the study participants were married 899 (95.6%). Majority of the study participants 900 (95.7%) were adults with a mean age and standard deviation of 26.6 (±4.59) years. Six hundred seventy-two (71.5%) of the study participants were housewife’s while 751(79.89%) of them were Orthodox Christianity followers. Likewise, 632(69%) reported their marital condition as “Good” and half of the study participants, 472 (50.2%), had low income. Five hundred ninety-two (62.98%) and 890 (94.7%) of the participants were in their 2^nd^ trimester and had access for at least one ANC visit in their current pregnancy, respectively. (Table 1)

**Table 1:** Socio-demographic and maternal characteristics of the study participants in Gondar town, northwest Ethiopia, 2018.

Variable	Number	Frequency (%)
**Age of mother**		
** Adolescent (18–19)**	40	4.26
** Adult (20–45)**	900	95.74
**Maternal education**		
** No formal education**	127	13.51
** Grade 1–8**	243	25.85
** Grade 9–12**	353	37.55
** Diploma and above**	217	23.09
**Maternal occupation**		
** Government employee**	129	13.72
** Housewife**	672	71.49
** Self-employee**	123	13.09
** Student**	16	1.70
**Maternal marital status**		
** Divorced**	6	0.64
** Married**	899	95.64
** Single**	18	1.91
** Separated**	17	1.81
**Marital condition**		
** Bad**	39	4.15
** Good**	901	95.85
**A religion of the mother**		
** Muslim**	180	19.15
** Orthodox**	751	79.89
** Protestant**	9	0.96
**Household income**		
** Low**	472	50.21
** Medium**	375	39.89
** High**	93	9.89
**ANC attendance**		
** Yes**	890	94.68
** No**	50	5.32
**Type of pregnancy**		
** Unwanted**	21	2.23
** Unplanned**	137	14.57
** Planned**	782	83.19
**Pregnancy time**		
** Second Trimester**	348	37.02
** Third Trimester**	592	62.98
**Parity**		
** 0**	358	38.09
** 1**	295	31.38
** 2–8**	287	30.53

### Nutrition and psychosocial health-related characteristics of the study participants

The prevalence of undernutrition among pregnant mothers using MUAC measurement was 14.4% (95%CI: 12.3–16.7). Mothers who had depression and anxiety based on EPDS score were 87(9.26%) and 112(11.91%), respectively. Reviewing the status of participants’ social support showed that 747(79.47%) of the pregnant mothers had good social support and parallel to this, 693 (75.2%) of the participants witnessed that their husband supported them always or most of the time. Among the participated pregnant mothers, 872(92.8%) were leveled their health condition as ‘‘Good” and most of them, 921(97.98), were reported as they were doing physical activity. In addition, 396 (42.2%), of the pregnant mothers explained that they have cups of coffee in a daily bases. (Table 2)

**Table 2:** Nutritional status and psychosocial related characteristics of the study participants in Gondar town, northwest Ethiopia, 2018.

Variables	Number	Frequency (%)
**Nutritional status**		
** Undernutrition**	135	14.4
** Normal**	805	85.6
**Food access for the last three months**		
** Yes**	888	94.47
** No**	52	5.53
**Depression**		
** Depressed**	87	9.26
** Normal**	853	90.74
**Anxiety**		
** Yes**	112	11.91
** No**	828	88.09
**Previous history of depression**		
** Yes**	69	7.34
** No**	871	92.66
**Social support**		
** Good support**	747	79.47
** Poor support**	193	20.53
**Husband support**		
** Always**	423	46.07
** Most of the time**	270	29.15
** Some of the time**	178	19.21
** Rarely/Never**	69	2.73
**Physical activity**		
** Yes**	921	97.98
** No**	19	2.02
**Coffee drinking**		
** Daily**	396	42.2
** Sometimes (1–3 times in a week)**	317	33.7
** Never**	227	24.1
**Maternal health condition**		
** Good**	872	92.77
** Bad**	68	7.23

### Factors associated with undernutrition

On a multivariable binary logistic regression analysis, after adjusting for other co-variables, the age of the mother, marital condition, and coffee drinking had a significant association with nutritional status of pregnant mothers. Accordingly, as the age of the pregnant mothers increases by one year, the odds of undernutrition decreases by 10% (AOR: 0.90; 95%CI: 0.87–0.95). Those pregnant mothers who had a bad marital condition (AOR: 2.18; 95%CI: 1.03–4.59) were 2.18 times more likely to be undernourished than those mothers who had a good marital condition. Mothers who drink coffee one to three days per week were 43% less likely to develop under-nutrition as compared to pregnant mothers who drink coffee daily. (Table 3)

**Table 3:** Factors associated with nutritional status of pregnant mothers in Gondar town, northwest Ethiopia, 2018.

Variable	Nutritional status		COR_95%CI_	AOR_95%CI_
	Under-nutrition	Normal		
**Age of the mother**	135	805	0.91(0.87,0.95)	0.90(0.87,0.95)*
**Marital status**				
** Married**	125	774	1.99(0.95,4.17)	
** Divorced/separated**	10	31	1	
**Marital condition from a wife's perspective**				
** Good**	124	777	1	1
** Bad**	11	28	2.46(1.19,5.07)	2.18(1.03,4.59)*
**Household income**				
** Low**	79	393	1	
** Medium**	47	328	0.71(0.48,1.05)	
** High**	9	84	0.53(0.26,1.10)	
**Type of pregnancy**				
** Planned**	107	675	1	
** Unplanned**	23	114	1.50(0.67,3.34)	
** Unwanted**	5	16	4.04(1.13,14.43)	
**Mothers education**				
**No formal education**	21	106	1.39(0.75, 2.58)	
** Grade 1–8**	45	198	1.59((0.95,2.68)	
** Grade 9–12**	42	311	0.95(0.57,1.59)	
** Diploma and above**	27	190	1	
**Difficult to access food in the last three months**				
** Yes**	11	41	1.65(0.83,3.30)	
** No**	124	764	1	
**Depression**				
** Yes**	14	73	1.16(0.63,2.12)	
** No**	121	732	1	
**Anxiety**				
** Yes**	15	97	1.20(0.61,1.95)	
** No**	120	708	1	
Anxiety*Depression	P-value (0.63)			
**Husband support**				
** Always**	96	597	1	
** Some of the time**	24	154	0.97(0.59,1.56)	
** Rarely/Never**	15	54	1.73(0.94,3.18)	
**Parity**				
** 0**	63	295	1	
** 1**	38	257	0.69(0.45,1.07)	
** 2–8**	34	253	0.63(0.40,0.98)	
**Coffee drinking**				
** Daily**	65	331	1	
**Sometimes (1–3 times per week)**	34	283	0.61(0.39,0.95)	0.57(0.36,0.89)*
** Never**	36	191	0.96(0.61,1.45)	0.96(0.61,1.50)

*significance at p-value <0.05

## Discussion

Maternal nutrition prior to and during pregnancy play a central role in determining the long-term health and nutritional effect of both the mother and her growing fetus [9, 21]. Maternal undernutrition is highly prevalent in low and middle-income countries [7, 9, 10] and Ethiopia as one of these countries has been significantly affected by the burden of undernutrition. We conducted a cross-sectional study to identify the burden and associated factors of undernutrition among pregnant mothers residing in an urban setting. The burden of maternal undernutrition in Gondar town was 14.4%. This finding was similar to the study conducted in the same place with the current study that reported 16.2% [8]. The insignificant discrepancy might be due to the study setup, the current study was community-based while the previous was an institution based study, as institution-based studies might overestimate the true magnitude of the problem.

Compared to our finding, a lower prevalence of undernutrition was reported in Wondo Genet (9.2%) [22] that used a different cut off value with our study; they have used a MUAC <21cm while we have used a MUAC <22cm. The other possible source of discrepancy might be the term of pregnancy, we have included women in their second and third trimester while they have included all terms as pregnancy term could advance nutritional depletion. Likewise, three studies reported from Sudan [23], Nigeria [24] and Lebanon [25] have reported a relatively lower prevalence than ours and the possible reason might be the use of different measurement in addition to different socio-demographic nature of the study area; they have used BMI while we used MUAC.

On the contrary, the prevalence reported in the current study was far lower than a prevalence reported in Southern Nations Nationalities and People Region (SNNPR) 71.15 [26], Sidama, Southern Ethiopia (31.4%) [27], Gambella 28.6% [28], rural Eastern Ethiopia 19.8% [29] and Rayitu Woreda, Oromia 24% [14]. These studies were conducted in rural areas unlike ours that exclusively conducted in urban area.

Age of the mother, marital condition, and coffee drinking were showed a statistically significant association with undernutrition of pregnant mothers. Based on this study, the age of pregnant mothers was found conversely associated with the nutritional status of mothers. As the age of the mother increased by one year her risk of undernutrition decreases by 10% and it is consistent with a study conducted in southern Ethiopia [27], Lebanon [25] and Bangladesh [30]. This is because young mothers, apart from insufficient development of their reproductive system and their need of nutrition for their growing body, they are often remarkably surrounded by unfavorable nutritional conditions [21, 25, 31].

A marital condition was found to be an important predictor of pregnant mothers’ nutritional status. The probability of being undernourished was 2 times higher among pregnant mother who had a bad marital condition as compared to those mothers who had a good marital condition. A bad marital condition that explained as dissatisfaction in a marital relationship would affect a support or care that the mother should get from her partner and this could indirectly affect her nutritional condition.

We found that a frequent coffee drinking increased the risk of undernutrition. Pregnant mothers who none-frequently drink coffee were 43% less likely to develop malnutrition compared to those reported daily consumption of coffee. This is scientifically evidenced that moderate caffeine intake (300 mg/day) or taking about three cups of coffee is probably not harmful to pregnant mothers [32, 33] but taking a large amounts of caffeine may cause nutrient depletion and interfere with nutrient absorption [32–34]. In Ethiopian culture, coffee ceremony is conducted at least two times in a day (morning and night) and at each ceremony a minimum of three cups of coffee are taken right after meals and this would most affect nutrient absorption. Similar to other studies [35, 36], undernutrition was failed to have a significant association with depression and anxiety scale of pregnant mothers.

As a limitation; a cross-sectional nature of the study might affect the establishment of a causal relationship between identified risk factors and undernutrition as all the identified exposures/risk factors have a probability to change overtime; fail to incorporate a dietary diversity score as a risk factors of undernutrition might have also introduced a residual confounding problem.

## Conclusion

Although undernutrition in pregnant mothers in this study was found to be lower as compared to other similar studies, it should be considered as a major public health problem as undernutrition in pregnancy plays a key role for enhancing maternal health and child development. Our study also identified pregnant mothers at higher risk of being malnourished: younger age, those with a poor marital condition, and those reported a frequent coffee drinking.

The prevention of maternal undernutrition is a long-term investment and it requires a multi-sectoral collaboration and coordination between national and international organizations. Consequently, the concerned governmental and the existing non-governmental bodies shall strengthen their coordinated effort towards improving maternal nutrition by giving due consideration to pregnant mothers in younger age and those having a problem with their partner. Dual counseling service for the pregnant mothers and her partner during the antenatal care service might also helpful to enhance the support that the mother got to improve her nutritional status. The mothers shall also be counseled about the effect of frequent coffee consumption to their fetus and their health.

## Supporting information

S1 Table English version questioner to assess underweight and its associated factors in Gondar Town, Northwest Ethiopia. (DOCX)

S2 Table Amharic version questioner to assess underweight and its associated factors in Gondar Town, Northwest Ethiopia. (DOCX)

## Abbreviations

AOR: Adjusted odds ratio
95% CI: 95% Confidence Interval
COR: Crude Odds Ratio
EDHS: Ethiopian Demographic and Health Survey
MUAC: Mid-Upper Arm Circumference
NCDs: None Communicable Diseases
NNP II: National Nutrition Program II
