# Supplementary material for: Undernutrition and its associated factors among pregnant mothers in Gondar town, Northwest Ethiopia
Source: PLoS One. 2019 Apr 22;14(4):e0215305. doi: 10.1371/journal.pone.0215305 (PMC6476509; doi:10.1371/journal.pone.0215305)
Supplement: S1 Table — (DOCX) [file pone.0215305.s001.docx]

# Annex 4: Questionnaire for collecting quantitative data

English version questioner to assess underweight and its associated factors in Gondar Town, Northwest Ethiopia.

## Annex 4a: Questionnaire for collecting quantitative data

## Time required: 35’-40’

Kebele name: ---------------------------------------------------------

Code of the mother: --------------------------------------------------

Name of the interviewer: ------------------------------------------------

Date of the interview: -------------------------------------------------------

Date of appointment for next visit -----------------------------------------

**Direction**: Please circle the response options and clearly fill open ended questions

| **Code** | **Variable** | **Response** | **Score/ explain** |
| --- | --- | --- | --- |
| **Part I**: **Socio-demographic characteristics of the women, now I will ask you about your self** | | | |
| 1.01 | Age of the mother | ------- years |  |
| 1.02 | Educational status of the mother | 1. No formal education 2. Grade 1-8 3. Grade 9-12 4. Diploma and above |  |
| 1.03 | Occupation of the mother | 1. Housewife 2. Student 3. Government employee 4. Self-employee |  |
| 1.04 | Marital status of the mother | 1. Single 2. Married 3. Divorced 4. Widowed 5. Separated |  |
| 1.05 | How you can explain your marital condition in general? | 1. Very good 2. Good 3. Bad 4. Very Bad |  |
| 1.06 | How often you discuss and agree with your husband on day to day life? | 1. Most of the time 2. Sometimes 3. Rarely 4. Never |  |
| 1.07 | Are you an active follower of any religion? | 1. Orthodox 2. Muslim 3. Catholic 4. Protestant 5. Other |  |
| 1.08 | In the last three months, have you ever worried that your household would not have enough food? | 1. Yes 2. No |  |
| 1.09 | Your monthly income in Ethiopian birr | -------------- |  |
| **Part II: Fertility: Now, I would like to ask you about the births that you have had in your life.** | | | |
| 2.01 | How many children do you have? | ------------ children |  |
| 2.02 | Is your current pregnancy a planned pregnancy? | 1. Yes, I wanted a child at this time 2. No, but I did want a child - later 3. No, I did not intend to have a child at all |  |
| 2.03 | How many months pregnant are you now? | -------------- weeks |  |
| 2.04 | Did you have difficulty in getting pregnant this time (e.g. used any fertility medication/waited long time to get pregnant? | 1. Yes 2. No |  |
| 2.05 | Mid upper arm circumference(MUAC) of the mother | -----------mm |  |
| **Part III. Feelings of depression (EPDS)**. **Tell us the way you have been feeling in the past (1) week including today. In the past seven days,** | | | |
| 3.01 | In the last week, have you been able to laugh and see the funny side of things? | As much as I always used to  Not as much as I used to  Certainly not as much as I used to  Not at all | 0  1  2  3 |
| 3.02 | In the last week, have you looked forward with enjoyment to things? | As much as I always used to  Rather less  Certainly less  Never looked forward | 0  1  2  3 |
| 3.03 | In the last week, have you blamed yourself unnecessarily when things went wrong? | Most of the time  Sometimes  Rarely  Never | 3  2  1  0 |
| 3.04 | In the last week, have you been anxious or worried for no good reason? | Most of the time  Sometimes  Not often  Never | 0  1  2  3 |
| 3.05 | In the last week, have you felt scared or panicky for no good reason? | Most of the time  Sometimes  Rarely  Never | 3  2  1  0 |
| 3.06 | In the last week, have things been getting on top of you? | Most of the time unable to cope  Sometimes unable  Mostly able  Coping as usual | 3  2  1  0 |
| 3.07 | In the last week, have you been so unhappy that you have had difficulty sleeping? | Most of the time  Sometimes  Rarely  Never | 3  2  1  0 |
| 3.08 | In the last week, have you felt sad or miserable? | Most of the time  Sometimes  Occasionally  Never | 3  2  1  0 |
| 3.09 | In the last week, have you felt so unhappy that you have been crying? | Most of the time  Sometimes  Occasionally  Never | 3  2  1  0 |
| 3.10 | In the last week, has the thought of harming yourself occurred to you? | Frequently  Sometimes  Not often  Never | 3  2  1  0 |
| 3.11 | Have you felt these symptoms before being pregnant? | 1. Yes 2. No |  |
| **Part IV. Oslo Social Support Scale (OSSS-3) to assess pregnant women social support,** Now, we would like to ask you questions about the support you get from different people | | | |
| 4.01 | How many people are so close to you that you can count on them if you have serious personal problems? | None  1 or 2  3 to 5  6 or more | 1  2  3  4 |
| 4.02 | How much concern do people show in what you are doing? | A lot of concern and interest  Some concern and interest  Uncertain  Little concern and interest  No concern and interest | 5  4  3  2  1 |
| 4.03 | How easy is it to get practical help from neighbours if you should need it? | Very easy  Easy  Possible  Difficult  Very difficult | 5  4  3  2  1 |
| 4.04 | My husband helps me a lot | Always  Most of the time  Some of the time  Rarely  Never | 5  4  3  2  1 |
| **Part V. Obstetric related factors, now I would like to ask you questions related to your current pregnancy** | | | |
| 5.01 | After you knew that you are pregnant, did you go anywhere to receive antenatal care? | 1. Yes 2. No |  |
| 5.02 | Including this pregnancy, for how many times have you been pregnant and give birth | 1. For the first time 2. Two and above | If “1” got Q. No 5.05 |
| 5.03 | Have you ever given birth to low weight baby? | 1. Yes 2. No |  |
| 5.04 | Have you ever given birth to preterm? | 1. Yes 2. No |  |
| 5.05 | Have you ever had a baby by cesarean delivery | 1. Yes 2. No |  |
| 5.06 | Do you have any fear of giving to this birth? | 1.Yes  2.No |  |
| 5.07 | Are you and your husband interested in the sex of your current pregnancy? | 1.Yes  2.No |  |
| 5.08 | Have you practice physical activity such as brisk walking, dancing, gardening, and usual housework for at least three hours/week | 1. Yes 2. No |  |
| 5.09 | How do you rate your daily health condition? | 1. Very good 2. Good 3. Bad 4. Very bad |  |
| 5.10 | Is there anybody who smoke near to you in your home or in your work place? | 1. Yes (exposure to second hand smoking) 2. No |  |
| 5.11 | How often are you drinking coffee during this pregnancy? | 1. Daily 2. Sometimes 3. Never |  |
| **Part VI. Stress coping ability of the women by Perinatal Coping Inventory(PCI-4)** | | | |
| 6.01 | Planned how you will handle the birth | Frequently  Sometimes  Not often  Never | 3  2  1  0 |
| 6.02 | Felt that being pregnant has enriched your life | Frequently  Sometimes  Not often  Never | 3  2  1  0 |
| 6.03 | Prayed that the birth will go well | Frequently  Sometimes  Not often  Never | 3  2  1  0 |
| 6.04 | Avoided being with people in general | Frequently  Sometimes  Not often  Never | 0  1  2  3 |
