# Supplementary material for: Undernutrition and its associated factors among pregnant mothers in Gondar town, Northwest Ethiopia
Source: PLoS One. 2019 Apr 22;14(4):e0215305. doi: 10.1371/journal.pone.0215305 (PMC6476509; doi:10.1371/journal.pone.0215305)
Supplement: S2 Table — (DOCX) [file pone.0215305.s002.docx]

Amharic version questioner to assess underweight and its associated factors in Gondar Town, Northwest Ethiopia.

የቀበሌው ስም---------------------

የእናትዬው መለያ ቁጥር ------------------

የቃለመጠይቅ አድራጊው ስም-------------------------------------------------

ቃለ መጠይቅ የተደረገበት ቀን--------------------------------------

| **መለያ** | **መጠይቅ** | **መልስ** |  |
| --- | --- | --- | --- |
| **የመጀመርያ ክፍል፤ የእናቶች አጠቃላይ መሃበራዊ መረጃ** | | | |
| 1.01 | የእናትየው እድሜ | ------------ዓመት |  |
| 1.02 | የእናትየው የት/ት ደረጃ | 1. መደበኛ ት/ት ያልተከታተሉ 2. ክፍል 1-8 3. ክፍል 9-12 4. ዲፕሎማ ና ከዝያ በላይ |  |
| 1.03 | የእናትየው የስራ ሁኔታ | 1. የቤት እመቤት 2. ተማሪ 3. የመንግስት ሰራተኛ 4. የግል ሰራተኛ |  |
| 1.04 | የእናትየው የጋብቻ ሁኔታ | 1. ያላገባች 2. ያገባች 3. የተፋታች 4. የሞተባት 5. ተለያይታ የምትኖር |  |
| 1.05 | በለተለት ከባለቤትዎ ጋር በሚያደርጉት የቤት ውስጥ ውይይት ለምንያክል ጊዜ ለስምምነት ይደርሳሉ; | 1. አብዛኛውን ጊዜ 2. አንዳንድ ጊዜ 3. በጣም ትንሽ ጊዜ 4. አንድም ቀን ለስምምነት ደርሰን አናውቅም |  |
| 1.06 | የየትኛው የሀይማኖት ተከታይ ኖት | 1. አርቶዶክስ 2. ሙስሊም 3. ካቶሊክ 4. ፕሮቴስታንት |  |
| 1.07 | በባለፉት ሶስት ወራት ውስጥ፤ ቤተሰቦቼ የሚበሉትን በቂ ምግብ ሊያጡ ይችላሉ ብለው ፈርተው ያውቃሉ;; | 1. አዎ 2. አይ |  |
| 1.08 | ወራዊ ገገቢዎ ምንያክል ይሆናል ብለው ይገምታሉ | ---------- |  |
| ክፍል ሁለት፡ በዚህ ክፍል በህይወትዎ ዘመን ስላፈሯቻቸው ልጆች የሚለከት ጥያቀዎች እጠይቆታለው | | | |
| 2.01 | ባጠቃላይ ምን ያክል ልጆች አፍርተዋል; | --------- |  |
| 2.02 | ያሁኑ እርግዝናዎ የታቀደ ነበር; | 1. አዎ፤ አሁን መውለድ ፈልጌ ነው 2. አይደለም፤ ነገር ግን ቆይቼ መወለድ እፈልግ ነበር 3. አይደለም፤ ባጠቃላይ ልጅ እንዲኖረኘኝ አልፈልግም ነበር |  |
| 2.03 | የስንት ወር ነብሰጡር ነሽ አሁን; | ---------- |  |
| 2.04 | ይህን እርግዝና ለማግኘት በጣም ተቸግረው ነበር፤ ለምሳል፤ ረዥም ጊዜ ተብቀው ነበር፤ ለምርመራ ሄደው ነበር | 1. አዎ 2. አይደለም |  |
| 2.05 | የእናትየው የ MUAC ልኬታ | -----------ሚሜ |  |
| **አክፍል ሶስት፤ አሁን የሚሰማዎትን ስሜት ብቻ ሳይሆን፡ በባለፉት 7 ቀናት የተሰማዎት ስሜት ካለ ከዚያ ስሜት ጋር የሚጠጋውን አይነት ከተሰጥዎት ምርጫ ውስጥ ይመረጡ** | | | |
| 3.01 | በባለፈው ሳምንት፤ስቄአለሁም አስቂኝ ሁኔታዎችንም መለየት ችያለሁ፡ | በፊት ሳደርግ እንደነበረው  እንደበፊቱ አይሆንም  በጭራሽ እንደበፊቱ አይሆንም  በጭራሽ አልስቅም | 0  1  2  3 |
| 3.02 | በባለፈው ሳምንት፤መጪውን ሁኔታ በደስታ መቀበል እችላለሁ፡ | በፊት የማደርገውን ያህል  በፊት ከማደርገው አነስ ያለ  በእርግጥ በፊት ከማደርገው ያነስ  በጭራሽ አልችልም | 0  1  2  3 |
| 3.03 | በባለፈው ሳምንት፤ሁኔታዎች ሳይሳኩ ከቀሩ እራሴን እወቅሳለሁ፡ | አዎን፤ ሁልጊዜ  አዎን፤ አልፎ አልፎ  እስከዚህም እራሴን አልወቅስም  በጭራሽ እራሴን አልወቅስም | 3  2  1  0 |
| 3.04 | በባለፈው ሳምንት፤በማይረባ ነገር እጨነቃለሁ፤ አጠበባለሁ፡ | በጭራሽ  ከቁጥር ለማይገባ ጊዜ  አዎን፤ አልፎ አልፎ  አዎን፤ ሁልጊዜ | 0  1  2  3 |
| 3.05 | በባለፈው ሳምንት፤በማይረባ ምክንያት ፍርሃትና ድንጋጤ ይሰማኛል፡ | አዎን፤ ሁልጊዜ  አዎን፤ አልፎ አልፎ  እስከዚህ አይሰማኝም  በጭራሽ አይሰማኝም | 3  2  1  0 |
| 3.06 | በባለፈው ሳምንት፤ሁኔታዎች ተደራርበውብኛል፡ | አዎን፤ አብዛኛውን ጊዜ ሁኔታዎችን መቋቋም አልችልም  አዎን፤ አልፎ አልፎ ሁኔታዎችን መቋቋም አልችልም  አይ፤ አብዛኛውን ጊዜ ሁኔታዎችን መቋቋም ችያለሁ  አይ፤ በፊት እንደማደርገው ሁኔታዎችን መቋቋም ችያለሁ | 3  2  1  0 |
| 3.07 | በባለፈው ሳምንት፤ደስታ ከማጣቴ የተነሳ እንቅልፍ አይወስደኝም፡ | አዎን፤ ሁልጊዜ  አዎን፤ አልፎ አልፎ  እስከዚህም አልተቸገርኩም  በጭራሽ አልተቸገርኩም | 3  2  1  0 |
| 3.08 | በባለፈው ሳምንት፤ብስጭትና ሃዘን ተሰምቶኛል፡ | አዎን፤ ሁልጊዜ  አዎን፤ አልፎ አልፎ  እስከዚህ አይሰማኝም  በጭራሽ አይሰማኝም | 3  2  1  0 |
| 3.09 | በባለፈው ሳምንት፤ደስታ ከማጣቴ የተነሳ አለቅሳለሁ፡ | አዎን፤ አብዛኛውን ጊዜ  አዎን፤ በየጊዜው  አልፎ አልፎ ብቻ  በጭራሽ አላለቀስኩም | 3  2  1  0 |
| 3.10 | በባለፈው ሳምንት፤ራሴን የመጉዳት/የማጥፋት ሃሳብ ደርሶብኛል፡ | አዎን፤ ሁልጊዜ  አልፎ አልፎ ገጥሞኛል  እስከዚህም አልገጠመኝም  በጭራሽ አልገጠመኝም | 3  2  1  0 |
| 3.11 | ከላይ የተዘረዘሩት ስሜቶች ከማርገዝዎ በፊት ይሰማዎት ነበር | 1. አዎ 2. አይደለም |  |
| **ክፍል አራት፡ ከዚህ ቀጥሎ ስለሚየገኙት የማህበረሰብ ድጋፍ የሚመለከቱ ጥቄዎችን እተይቆታለሁ** | | | |
| 4.01 | ከባድ ችግር ቢገጥሞት በፍጥነት ሊደርሱሎት የሚችሉ ሰዎች ቁጥር ስንት ይሆናል ብለው ይገምታሉ | ማንም  1 ወይም 2  ከ 3 እስከ 5  6 ና ከዚያ በላይ | 1  2  3  4 |
| 4.02 | በሚያደርጉት እያነዳንዱ ነገር፤ ባከባቢው መሚገኙ ሰዎች ምን ያክል ትኩረት ያገኛሉ | ብዙ ትኩረት ና የመርዳት ፍላጎት ያላቸው ሰዎች አገኛለሁ  የተወሰነ ትኩረት ና የመርዳት ፍላጎት ያላቸው ሰዎች አገኛለሁ  እርግጠኛ አይደለሁም  ትንሽ ትኩረት ና የመርዳት ፍላጎት ያላቸው ሰዎች አገኛለሁ  ምንም አይነት ትኩረት ና የመርዳት ፍላጎት ያለቸው ሰዎች የሉም | 5  4  3  2  1 |
| 4.03 | እርዳታ ቢፈልጉ ምንያክል በተግባር ከጎረቤትዎ ማግኘት ይችላሉ | በጣም በቀላሉ ማግኘት እችላለሁ  በቀላሉ ማግኘት እችላለሁ  ማግኘት እችላለሁ  ማግኘት ይከብዳል  ማግኘት በጣም ይከብዳል | 5  4  3  2  1 |
| 4.04 | ባለቤቴ በሚችለው ሁሉ እያገዘኝ ና እያበረታታኝ ነው | ሁልጊዜ  ብዙዉን ጊዜ  አነዳንድ ጊዜ  እምብዛም አይደለም  ምንም አይነት እገዛ አያደርግልኝም | 4  3  2  1  0 |
| **ክፍል አምስት፡ ከዚህ ቀጥሎ ባጠቃላይ ስለ አሁን እርግዝናዎ እጠይቆታለው** | | | |
| 5.01 | ነብሰ ጡር መሆንዎን ካወቁ በኋላ ለእርግዝና ምርመረ ለማድረግ ወደ ጤና ተቋም ሄደው ያውቃሉ | 1. አዎ 2. አልሄድኩም |  |
| 5.02 | ይሄን እርግዝና ጨምሮ ለስንት ጊዜ አርግዘው ወልደዋል | 1. ለ አንድ ጊዜ 2. ሁለት ና ከዚያ በላይ | U`Ý¨< 1 ŸJ’ ¨Å Øo lØ` 5.05 Ã´KK< |
| 5.03 | ከክብደት በታች የሆነ ልጅ ወልደው ያውቃሉ | 1. አዎ 2. አላውቅም |  |
| 5.04 | የመውለጃ ጊዜሽ ሳይደር በድንገት ወልደው ያውቃሉ | 1. አዎ 2. አላውቅም |  |
| 5.05 | በ ኦፕሬሽን ወልደው ያውቃሉ | 1. አዎ 2. አላውቅም |  |
| 5.06 | ከዚህ እርግዝና ና ውለደት ጋር ተያይዞ የሚሰማዎት ፍራት ወይም ስጋት አለ | 1. አዎ 2. የለም |  |
| 5.07 | በእርስዎ ና በባለቤትዎ መካከል በተጸነሰው ልጅ የጾታ ፍላጎት ልዩነት አለ | 1. አዎ 2. የለም |  |
| 5.08 | በባለፉት የእርግዝና ወራት አካል ብቃት እንቅስቃሴ (መደነስ፤ረዥም የእግር እንቅስቃሴ፤ አትክልት ማጠጣት እና ሙሉ የቤት ውስጥ ስራን መሸፈን) ያደርጉ ነበር | 1. አዎ 2. አላደረኩም |  |
| 5.09 | የእለት ከ እለት የጤና ሁኔታዎን እነዴት ይመዝኑታል | 1. በጣም ደህና ነበርኩ 2. ደህና ነበርኩ 3. ጥሩ አልነበርኩም 4. በጣም ጥሩ አልነበርኩም |  |
| 5.10 | በአከባቢዎት (በቤትዎ ወይም በስራ ቦታዎ ወይም በጎረቤትዎ) ሲጋራ የሚያጨስ ሰው አለ | 1. አዎ 2. የለም |  |
| 5.11 | ቡና ምንያክል ይጠጣሉ | 1. በየቀኑ 2. አንዳንድ ጊዜ 3. ጠጥቼ አላውቅም |  |
| **ክፍል ስድስት፤ ከዚህ በመቀጠል ውጥረት የመከላለከልን የሚመለከቱ ጥያቄዎችን እንጠይቆታለን** | | | |
| 6.01 | ልጅዎትን እነዴት ና በምን ሁኔታ እንደምትወልዱ አቅደዋል/አስበዋል | ሁሌም አስባለሁ  አንዳነዴ አስባለሁ  ብዙ ጊዜ አላስብም  አስቤውም አላውቅም | 3  2  1  0 |
| 6.02 | ማርገዜ ህይዎቴን ና አኗኗሬን ይቀይርልኛል ብለው ያስባሉ | ሁሌም አስባለሁ  አንዳነዴ አስባለሁ  ብዙ ጊዜ አላስብም  አስቤውም አላውቅም | 3  2  1  0 |
| 6.03 | እርግዝናዬ ና ውልደቴ ጥሩ እንዲሆን እየጸለዩኩ ነው | ሁሌም እጸልያለሁ  አንዳነዴ እጸልያለሁ  ብዙ ጊዜ እጸልያለሁ  ጸልዬ አላውቅም | 3  2  1  0 |
| 6.04 | ከሰው ጋር መሆንን አስወግጃለሁ ወይም እጠላለሁ | ሁሌም እጠላለሁ  አንዳነዴ አጠላለሁ  ብዙ ጊዜ እጠላለሁ  አልጠለም | 0  1  2  3 |
